# Pan-Cancer Analysis Reveals That *E1A Binding Protein p300* Mutations Increase Genome Instability and Antitumor Immunity

**DOI:** 10.3389/fcell.2021.729927

**Published:** 2021-09-20

**Authors:** Zuobing Chen, Canping Chen, Lin Li, Tianfang Zhang, Xiaosheng Wang

**Affiliations:** ^1^Department of Rehabilitation Medicine, First Affiliated Hospital, College of Medicine, Zhejiang University, Hangzhou, China; ^2^Biomedical Informatics Research Lab, School of Basic Medicine and Clinical Pharmacy, China Pharmaceutical University, Nanjing, China; ^3^Cancer Genomics Research Center, School of Basic Medicine and Clinical Pharmacy, China Pharmaceutical University, Nanjing, China; ^4^Big Data Research Institute, China Pharmaceutical University, Nanjing, China

**Keywords:** pan-cancer, *EP300* mutations, genome instability, anti-tumor immunity, immunotherapy response

## Abstract

*E1A binding protein p300* (*EP300*) is mutated in diverse cancers. Nevertheless, a systematic investigation into the associations of *EP300* mutations with genome instability and antitumor immunity in pan-cancer remains lacking. Using the datasets from The Cancer Genome Atlas, we analyzed the correlations between *EP300* mutations and genome instability and antitumor immune response in 11 cancer types. Compared to *EP300*-wild-type cancers, *EP300*-mutated cancers had significantly higher tumor mutation burden (TMB) in 10 cancer types. *EP300*-mutated cancers harbored a much higher fraction of microsatellite instable cancers in the colon and gastric cancers. *EP300* was co-mutated with genes involved in DNA damage repair pathways in multiple cancers. Furthermore, compared to *EP300*-wild-type cancers, *EP300*-mutated cancers had significantly higher immune cytolytic activity scores and ratios of immune-stimulatory over immune-inhibitory signatures in diverse cancers. Also, *EP300*-mutated cancers showed significantly higher programmed death-ligand 1 (*PD-L1*) expression levels than *EP300*-wild-type cancers. The increased TMB, antitumor immune activity, and PD-L1 expression indicated a favorable response to immune checkpoint inhibitors (ICIs) in *EP300*-mutated cancers, as evident in three cancer cohorts treated with ICIs. Thus, the *EP300* mutation could be a predictive biomarker for the response to immunotherapy.

## Introduction

*E1A binding protein p300* (*EP300*) is a gene encoding a histone acetyltransferase, which is involved in chromatin remodeling to regulate the transcription of numerous genes (Eckner et al., 1994). The EP300 protein plays an essential role in regulating cell proliferation and differentiation (Gayther et al., 2000). Consequently, the mutation of *EP300* has correlations with cancer development and prognosis (Bi et al., 2019; Huang et al., 2021). Indeed, this gene is mutated in various cancers (Sun et al., 2018), such as bladder cancer, cervical squamous cell carcinoma and endocervical adenocarcinoma (CESC), uterine corpus endometrial carcinoma (UCEC), lung cancer, melanoma, head and neck squamous cell carcinoma (HNSC), gastric cancer, and colorectal cancer. Previous studies have shown that *EP300* might act as a tumor suppressor gene (Asaduzzaman et al., 2017) or oncogene (Bi et al., 2019). Moreover, previous studies have shown that *EP300* mutations have associations with genome instability and antitumor immunity (Krupar et al., 2020; Zhu et al., 2020). For example, Zhu et al. (2020) showed that *EP300* mutations correlated with increased tumor mutation burden (TMB) and antitumor immunity in bladder cancer. Krupar et al. (2020) revealed that *EP300* mutations increased antitumor immunity *via* metabolic modulation.

Despite these previous studies, a systematic investigation into the associations of *EP300* mutations with genome instability and antitumor immunity in pan-cancer remains lacking. This study investigated the association between *EP300* mutations and genome instability in 11 cancer types from The Cancer Genome Atlas^1^ database. These cancer types included urothelial bladder carcinoma (BLCA), HNSC, skin cutaneous melanoma (SKCM), CESC, UCEC, stomach adenocarcinoma (STAD), lung adenocarcinoma (LUAD), breast invasive carcinoma (BRCA), liver hepatocellular carcinoma (LIHC), esophageal carcinoma (ESCA), and colon adenocarcinoma (COAD). We opted to analyze the 11 cancer types because each of them harbored more than 10 *EP300*-mutated tumor samples. We also investigated the association between *EP300* mutations and antitumor immune activity in these cancer types. Our study demonstrates that *EP300* mutations are associated with increased genome instability and antitumor immunity and thus is a predictive biomarker for the response to cancer immunotherapy.

## Materials and Methods

### Datasets

We attained gene expression (RSEM normalized) and somatic mutation profiling data for the 11 TCGA cancer types from the Genomic Data Commons (GDC) data portal.^2^ Also, we downloaded gene expression (RSEM normalized) and somatic mutation profiling data for human cancer cell lines from the Cancer Cell Line Encyclopedia (CCLE) project.^3^ In addition, we downloaded data of somatic mutations and drug sensitivity (IC50 values) of cancer cell lines to 192 antitumor compounds from the Genomics of Drug Sensitivity in Cancer (GDSC) project.^4^ Besides, we obtained somatic mutation profiling and clinical data for three melanoma cohorts treated with immune checkpoint inhibitors (ICIs) from their associated publications, including the Hugo et al. (2016), Riaz et al. (2017), and Liu and Schilling (2019) cohorts. A description of these datasets is shown in Supplementary Table 1.

### Gene Set Enrichment Analysis

To identify the Kyoto Encyclopedia of Genes and Genomes (KEGG) (Kanehisa et al., 2017) pathways highly enriched in *EP300*-mutated and *EP300*-wild-type pan-cancer of the 11 cancer types, we first identified the differentially expressed genes between *EP300*-mutated and *EP300*-wild-type pan-cancer by Student’s *t*-test using a threshold of false discovery rate (FDR) < 0.05 and fold change of mean expression levels >1.5. The FDR was the adjusted *p*-value evaluated by the Benjamini and Hochberg method (Benjamini and Hochberg, 1995). The differentially expressed genes included the upregulated genes in *EP300*-mutated pan-cancer and the upregulated genes in *EP300*-wild-type pan-cancer. By inputting the upregulated genes in *EP300*-mutated pan-cancer into the Gene Set Enrichment Analysis (GSEA) web tool (Subramanian et al., 2005), we obtained the KEGG pathways highly enriched in *EP300*-mutated pan-cancer with a threshold of FDR <0.05. Likewise, we obtained the KEGG pathways highly enriched in *EP300*-wild-type pan-cancer by inputting the upregulated genes in *EP300*-wild-type pan-cancer into GSEA.

### Network Analysis

We used BioGRID (Stark et al., 2006) to yield the protein–protein interaction network of EP300 by inputting the identifier “EP300,” selecting the organism “*Homo sapiens*” and using the default settings. BioGRID (version 4.3.195)^5^ is a database of protein, genetic, and chemical interactions. It includes 2,015,809 protein and genetic interactions, 29,093 chemical interactions, and 1,017,123 post-translational modifications from major model organism species based on 76,264 publications.

### Permutation Test

Because this study analyzed 11 cancer types, specific findings in some of these cancer types could be only by chance. We performed permutation tests by randomly exchanging class labels (*EP300*-mutated vs. *EP300*-wild-type) of tumor samples to explore whether our findings in a subset of these cancer types were statistically significant. In each permutation test experiment, we implemented 10,000 simulations.

### Statistical Analysis

We used Student’s *t*-test to compare two classes of normally distributed data, including gene expression levels, immune signature scores, and ratios of immune-stimulatory/immune-inhibitory signatures. We used the Mann–Whitney *U* test to compare two classes of other data that were not normally distributed. We used Fisher’s exact test to explore the association between two categorical variables. All the statistical and computational analyses were performed in the R programming environment (version 3.6.1).

## Results

### *E1A Binding Protein p300* Mutations Correlate With Increased Genome Instability

**FIGURE 1:**
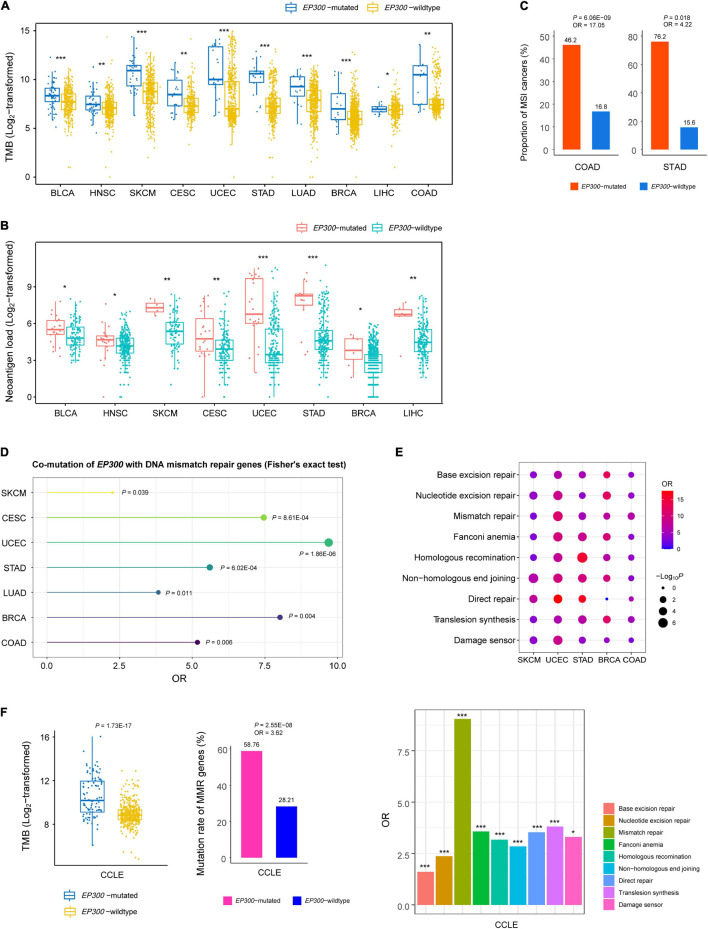
Associations between E1A binding protein p300 (*EP300*) mutations and genome instability. Comparisons of tumor mutation burden (TMB) **(A)**, neoantigens **(B)**, and proportions of microsatellite instability (MSI) cancers **(C)** between *EP300*-mutated and *EP300*-wild-type cancers. Co-mutation between *EP300* and seven DNA mismatch repair genes (*MLH1*, *MLH3*, *MSH2*, *MSH3*, *MSH6*, *PMS1*, and *PMS2*) **(D)** and DNA damage repair (DDR) pathway genes **(E)**. **(F)** Comparisons of TMB and mutation rates of DNA mismatch repair genes between *EP300*-mutated and *EP300*-wild-type cancer cell lines and comparisons of mutation rates between pathway-gene-mutated (PGM) and pathway-gene-wild-type (PGW) groups for each of the nine DDR pathways (Knijnenburg et al., 2018). The one-tailed Mann–Whitney *U* test *p*-values are shown in **(A,B)**. The *p*-values of Fisher’s exact test and odds ratio (OR) are shown in **(C–F)**. *P < 0.05, **P < 0.01, ***P < 0.001.

Genome instability may cause high TMB (Abbas et al., 2013). We compared TMB, defined as the total number of somatic mutations, between *EP300*-mutated and *EP300*-wild-type cancers. Interestingly, in 10 of the 11 cancer types (except ESCA), *EP300*-mutated cancers had significantly higher TMB than that in *EP300*-wild-type cancers (one-tailed Mann–Whitney *U* test, *p* < 0.05) (Figure 1A). Gene mutations may yield neoantigens recognized by immune cells. We found that *EP300*-mutated cancers showed more neoantigens (Rooney et al., 2015) than *EP300*-wild-type cancers in eight cancer types (*p* < 0.05) (Figure 1B). Among the 11 cancer types, UCEC, COAD, and STAD are prevalent with the microsatellite instability (MSI) subtype. We found that *EP300*-mutated cancers harbored a significantly higher proportion of MSI cancers than *EP300*-wild-type cancers in COAD and STAD [Fisher’s exact test, *p* < 0.05, odds ratio (OR) > 4] (Figure 1C). Moreover, in seven cancer types (SKCM, CESC, UCEC, STAD, LUAD, BRCA, and COAD), *EP300* was co-mutated with at least one of the seven genes (*MLH1*, *MLH3*, *MSH2*, *MSH3*, *MSH6*, *PMS1*, and *PMS2*) functioning in DNA mismatch repair (*p* < 0.05, OR > 2) (Figure 1D). The permutation test indicated that this result was statistically significant (*p* < 0.0001). In addition, we analyzed associations between *EP300* mutations and nine pathways involved in DNA damage repair (DDR) (Knijnenburg et al., 2018). The nine DDR pathways included base excision repair, nucleotide excision repair, mismatch repair, Fanconi anemia, homologous recombination, non-homologous end-joining, direct repair, translesion synthesis, and damage sensor. We divided each cancer type into pathway-gene-mutated (PGM) and pathway-gene-wild-type (PGW) groups for each of the nine DDR pathways. The PGM indicates at least a functionally deleterious mutation in the pathway genes, and the PGW indicates no functionally deleterious mutations in the pathway genes. Strikingly, we found that the PGM group harbored a significantly higher proportion of *EP300*-mutated cancers than the PGW group for all the nine pathways in SKCM, UCEC, and STAD (Figure 1E). Again, the permutation test revealed that this result was statistically significant (*p* < 0.0001). In addition, in BRCA and COAD, the PGM group included a significantly higher proportion of *EP300*-mutated cancers than the PGW group for seven of the nine pathways. In BLCA, CESC, LUAD, LIHC, and ESCA, the PGM group encompassed a higher proportion of *EP300*-mutated cancers than the PGW group for at least one of the nine pathways.

Besides the DNA mismatch repair genes, *EP300* was co-mutated with a large number of other genes. In pan-cancer of the 11 cancer types, *EP300* was co-mutated with 16,012 genes (FDR < 0.05, OR > 1) (Supplementary Table 2). In addition, there were 261 genes that were co-mutated with *EP300* in at least five cancer types (FDR < 0.05, OR > 1) (Supplementary Table 3). Likewise, the permutation test revealed that this result was statistically significant (*p* < 0.0001). Again, these results suggest that *EP300* mutations have a significant association with genome instability in cancer.

In human cancer cell lines derived from the 11 cancer types,^6^ *EP300*-mutated cell lines had higher TMB than *EP300*-wild-type cell lines (*p* = 1.73 × 10^–17^) (Figure 1F). Moreover, we analyzed the association between *EP300* mutations and TMB in individual types of cell lines. We found that TMB was significantly higher in *EP300*-mutated than in *EP300*-wild-type cell lines from seven of the 11 cancer types (*p* < 0.05). The permutation test demonstrated that this result was significant (*p* < 0.0001). Furthermore, *EP300* was co-mutated with at least one of the seven DNA mismatch repair genes (*p* = 2.55 × 10^–8^, OR = 3.6) in the cancer cell lines. In addition, *EP300* mutations were more frequent in PGM than in PGW cell lines for all the nine DDR pathways (*p* < 0.05, OR > 1.5) (Figure 1F).

Taken together, these results indicate a significant association between *EP300* mutations and genome instability in cancer.

### *E1A Binding Protein p300* Mutations Correlate With Increased Antitumor Immune Activity and Immunotherapy Response

**FIGURE 2:**
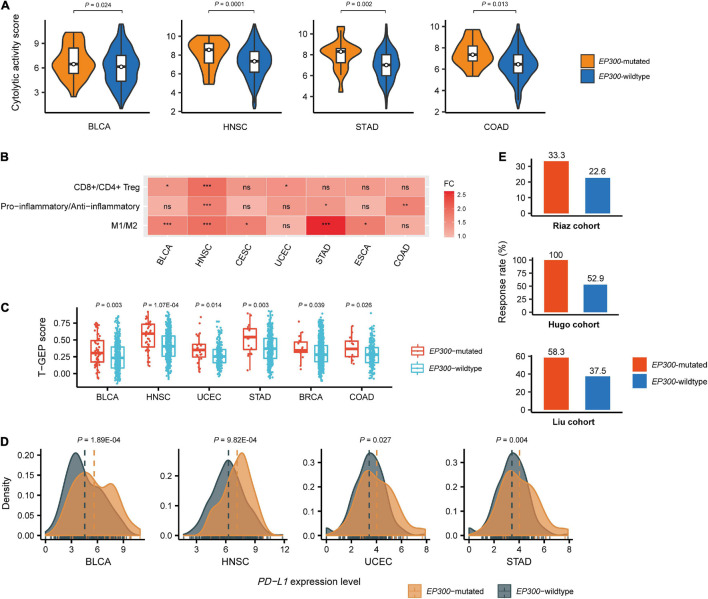
Associations between E1A binding protein p300 (*EP300*) mutations and antitumor immune signatures and immunotherapeutic response in cancer. Comparisons of immune cytolytic activity (CYT) scores **(A)**, the ratios of immune-stimulatory to immune-inhibitory signatures (CD8+/CD4+ regulatory T cells, pro-/anti-inflammatory cytokines, and M1/M2 macrophages) **(B)**, T cell-inflamed gene expression profile (T-GEP) scores **(C)**, programmed death-ligand 1 (*PD-L1*) expression levels, and the response rates to immune checkpoint inhibitors (ICIs) **(E)** in *EP300*-mutated and *EP300*-wild-type cancers. The CYT score is the average expression level of two marker genes (*GZMA* and *PRF1*) in the tumor (Narayanan et al., 2018). The ratio of immune-stimulatory to immune-inhibitory signatures is the log2-transformed geometric mean expression levels of the marker genes of immune-stimulatory signatures over those of immune-inhibitory signatures. The T-GEP score is the ssGSEA score of 18 T cell-inflamed genes (Ayers et al., 2017). The response rates to ICIs are for three melanoma cohorts receiving ICI treatments. The three cohorts included the Hugo et al. (2016), Riaz et al. (2017), and Liu and Schilling (2019) cohorts. The two-tailed Student’s *t*-test *p*-values are shown in **(A,B,D)**, and the one-tailed Mann–Whitney *U* test *p*-values are shown in **C**. *P < 0.05, **P < 0.01, ***P < 0.001, ns not significant.

We compared immune cytolytic activity (CYT) scores between *EP300-*mutated and *EP300*-wild-type cancers in the 11 cancer types. CYT represents the ability of cytotoxic T cells and natural killer cells to eliminate tumor cells and was used to assess antitumor immune response in cancer (Narayanan et al., 2018). The CYT score was defined as the average expression level of two CYT marker genes (*GZMA* and *PRF1*) in the tumor (Rooney et al., 2015). We found that CYT scores were significantly higher in *EP300-*mutated than those in *EP300-*wild-type cancers in four cancer types, including BLCA, HNSC, STAD, and COAD (two-tailed Student’s *t*-test, *p* < 0.05) (Figure 2A). Again, the permutation test demonstrated the significance of this result (*p* < 0.0001). Furthermore, we compared the ratios of immune-stimulatory over immune-inhibitory signatures in these cancer types. The ratios were log2-transformed geometric mean expression levels of the marker genes of immune-stimulatory signatures over those of immune-inhibitory signatures. We found that the ratios (CD8+/CD4+ regulatory T cells, pro-/anti-inflammatory cytokines, and M1/M2 macrophages) were significantly higher in *EP300*-mutated than those in *EP300*-wild-type cancers in multiple cancer types (two-tailed Student’s *t*-test, *p* < 0.05) (Figure 2B). For example, the ratios of M1/M2 macrophages were significantly higher in *EP300*-mutated than those in *EP300*-wild-type cancers in BLCA, HNSC, CESC, STAD, and ESCA; the permutation test showed that this finding was significant (*p* < 0.0001). These results suggest that *EP300* mutations are associated with increased antitumor immune activity in cancer.

We further compared a T cell-inflamed gene expression profile (T-GEP) between *EP300*-mutated and *EP300*-wild-type cancers. The T-GEP score was the ssGSEA score of 18 T cell-inflamed genes (Ayers et al., 2017). We found that T-GEP scores were significantly higher in *EP300*-mutated than in *EP300*-wild-type cancers in six cancer types, namely, BLCA, HNSC, UCEC, STAD, BRCA, and COAD (Figure 2C). Furthermore, we found that *EP300*-mutated cancers showed significantly higher programmed death-ligand 1 (*PD-L1*) expression levels than *EP300*-wild-type cancers in four cancer types, including BLCA, HNSC, UCEC, and STAD (Figure 2D). Because TMB, T-GEP, and PD-L1 expression were independent positive predictors for the response to ICIs (Cristescu et al., 2018) and elevated in *EP300*-mutated cancers, we expected that *EP300* mutations would correlate with a higher rate of response to ICIs. As expected, we found that the response rate was higher in *EP300*-mutated than that in *EP300*-wild-type cancers in three melanoma cohorts receiving ICI treatments. The three cohorts included the Hugo et al. (2016), Riaz et al. (2017), and Liu and Schilling (2019) cohorts. The response rates to ICIs in *EP300*-mutated vs. *EP300*-wild-type cancers were 33.3 vs. 22.6%, 100 vs. 52.9%, and 58.3 vs. 37.5% in the Hugo et al. (2016), Riaz et al. (2017), and Liu and Schilling (2019) cohorts, respectively (Figure 2E).

### Pathways Associated With *EP300* Mutations in Pan-Cancer

**FIGURE 3:**
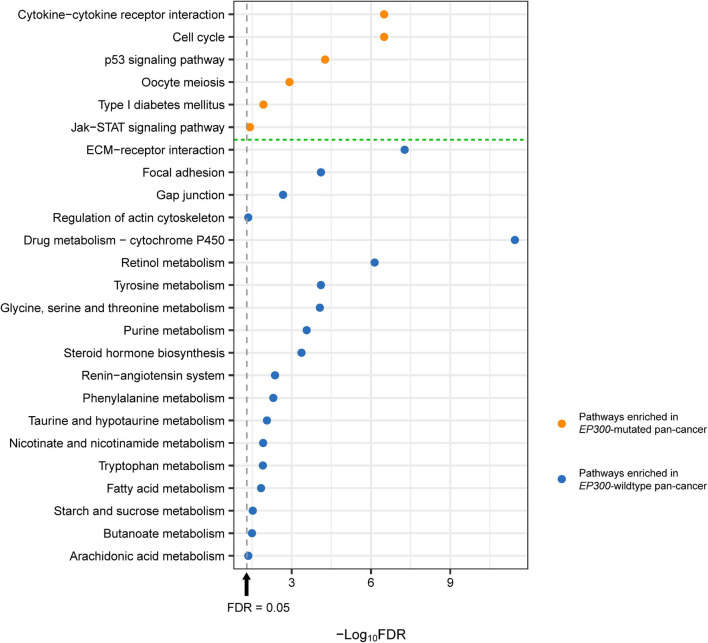
Kyoto Encyclopedia of Genes and Genomes (KEGG) pathways highly enriched in E1A binding protein p300 (*EP300*)-mutated and *EP300*-wild-type pan-cancer. These pathways were identified based on the differentially expressed genes between *EP300*-mutated and *EP300*-wild-type pan-cancer [Student’s *t*-test, false discovery rate (FDR) < 0.05, fold change (FC) > 1.5] by Gene Set Enrichment Analysis (GSEA) (Subramanian et al., 2005) with a threshold of FDR < 0.05. The FDR was the adjusted *p*-value evaluated by the Benjamini and Hochberg method (Benjamini and Hochberg, 1995).

We identified several KEGG (Kanehisa et al., 2017) pathways highly enriched in *EP300*-mutated vs. *EP300*-wild-type pan-cancer of the 11 cancer types by GSEA (Subramanian et al., 2005). The pathways highly enriched in *EP300*-mutated pan-cancer included cytokine–cytokine receptor interaction, cell cycle, p53 signaling, oocyte meiosis, type I diabetes mellitus, and Janus kinase (Jak)–signal transducer and activator of transcription (STAT) signaling (Figure 3). Among these pathways, cytokine–cytokine receptor interaction, type I diabetes mellitus, and Jak–STAT signaling were associated with immune signatures. Again, it suggests that *EP300* mutations are associated with increased antitumor immune activity in cancer. The significantly upregulated p53 signaling indicated the increased DDR activity in *EP300*-mutated pan-cancer since p53 plays a crucial role in DNA repair to maintain genome stability (Stegh, 2012). This is consistent with previous results of the significant association between *EP300* mutations and genome instability in cancer. In addition, the cell cycle pathway was also highly enriched in *EP300*-mutated cancers. It is justified since the aberrantly active cell cycle may contribute to genome instability in cancer (Abbas et al., 2013).

The pathways highly enriched in *EP300*-wild-type pan-cancer were mainly involved in the stromal signature and metabolic process. The stromal signature-associated pathways included extracellular matrix (ECM)–receptor interaction, focal adhesion, gap junction, and regulation of actin cytoskeleton. The metabolic process-associated pathways included drug metabolism–cytochrome P450; retinol metabolism; tyrosine metabolism; glycine, serine, and threonine metabolism; purine metabolism; renin–angiotensin system; phenylalanine metabolism; taurine and hypotaurine metabolism; nicotinate and nicotinamide metabolism; tryptophan metabolism; starch and sucrose metabolism; fatty acid metabolism; butanoate metabolism; arachidonic acid metabolism; and steroid hormone biosynthesis (Figure 3).

### Protein–Protein Interaction Network of EP300 in Cancer

**FIGURE 4:**
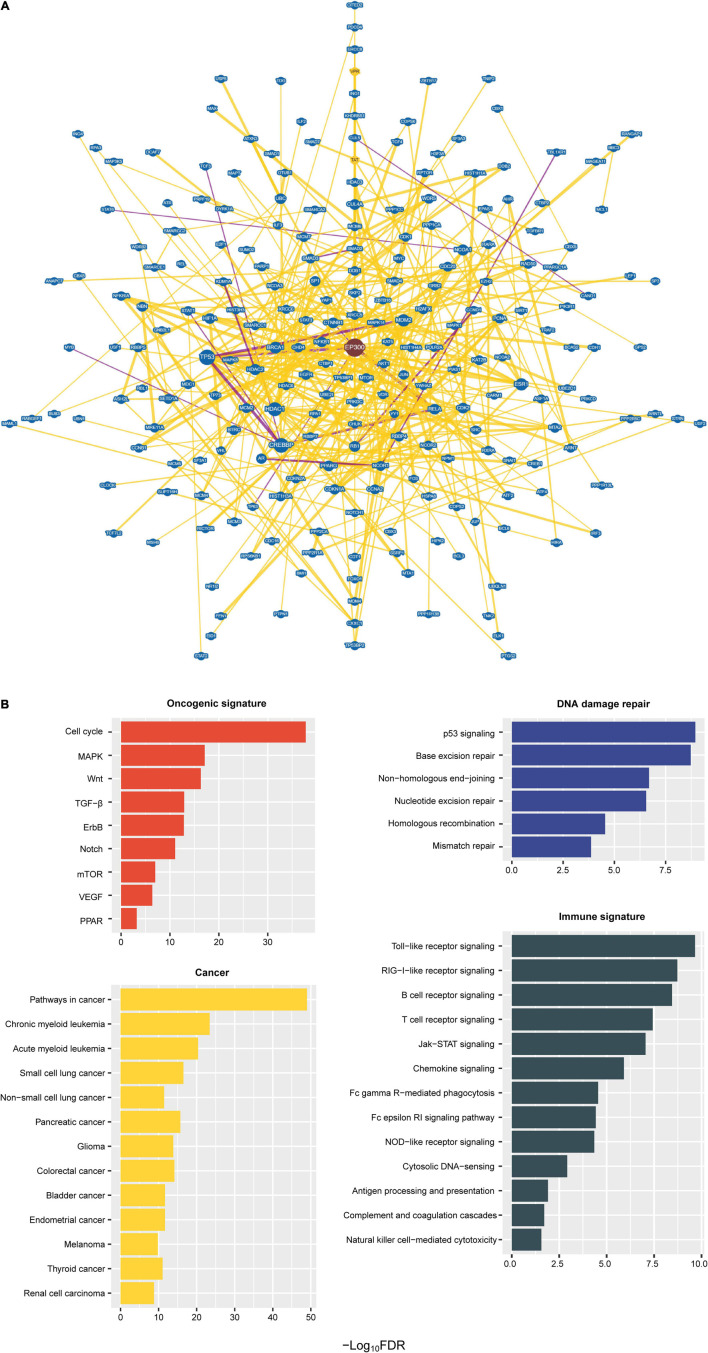
Protein–protein interaction network of E1A binding protein p300 (EP300). **(A)** A protein–protein interaction network of EP300 identified by BioGRID (Stark et al., 2006). The interactions evidenced by at least 10 publications are shown. **(B)** Kyoto Encyclopedia of Genes and Genomes (KEGG) pathways significantly associated with the 741 interactors of EP300 identified by Gene Set Enrichment Analysis (GSEA) (Subramanian et al., 2005) with a threshold of false discovery rate (FDR) < 0.05.

We identified a protein–protein interaction network of EP300 by BioGRID (Stark et al., 2006). A total of 741 proteins had an interaction with EP300 (Figure 4A and Supplementary Table 4). These proteins included numerous tumor suppressors, e.g., tumor protein p53 (p53), RB transcriptional corepressor 1 (RB1), BRCA1 DNA repair associated (BRCA1), cadherin 1 (CDH1), cyclin dependent kinase inhibitor 2A (CDKN2A), von Hippel-Lindau tumor suppressor (VHL), DNA damage recognition and repair factor (XPA), and SMAD family member 4 (SMAD4), and oncoproteins, e.g., epidermal growth factor receptor (EGFR), forkhead box O1 (FOXO1), forkhead box O3 (FOXO3), GATA binding protein 1 (GATA1), GATA binding protein 2 (GATA2), catenin beta 1 (CTNNB1), cyclin D1 (CCND1), bromodomain containing 4 (BRD4), CREB binding protein (CREBBP), Jun proto-oncogene, AP-1 transcription factor subunit (JUN), MDM2 proto-oncogene (MDM2), MDM4 regulator of p53 (MDM4), MYC proto-oncogene, bHLH transcription factor (MYC), MYB proto-oncogene, transcription factor (MYB), notch receptor 1 (NOTCH1), and RUNX family transcription factor 1 (RUNX1). These results suggest that EP300 dysfunction may play an essential role in tumor development. GSEA (Subramanian et al., 2005) identified 78 KEGG pathways significantly associated with the 741 interactors of EP300 (Figure 4B and Supplementary Table 5). These pathways were mainly involved in oncogenic signatures [e.g., cell cycle, mitogen-activated protein kinase (MAPK), Wnt, transforming growth factor (TGF)-β, ErbB, Notch, mammalian target of rapamycin (mTOR), vascular endothelial growth factor (VEGF), and peroxisome proliferator-activated receptor (PPAR)], DDR (e.g., p53 signaling, base excision repair, non-homologous end-joining, nucleotide excision repair, homologous recombination, and mismatch repair), and cancer (e.g., pathways in cancer, myeloid leukemia, lung cancer, pancreatic cancer, glioma, colorectal cancer, bladder cancer, endometrial cancer, melanoma, thyroid cancer, and renal cell carcinoma). In addition, many of the 78 pathways were involved in immune signatures, including Toll-like receptor signaling, RIG-I-like receptor signaling, B-cell receptor signaling, T-cell receptor signaling, Jak–STAT signaling, chemokine signaling, Fc gamma R-mediated phagocytosis, Fc epsilon RI signaling, NOD-like receptor signaling, cytosolic DNA-sensing, antigen processing and presentation, complement and coagulation cascades, and natural killer cell-mediated cytotoxicity. Again, these data suggest an essential role of EP300 in regulating various cellular and molecular processes in cancer.

### *E1A Binding Protein p300* Mutations Are Associated With Increased Drug Sensitivity in Cancer

**FIGURE 5:**
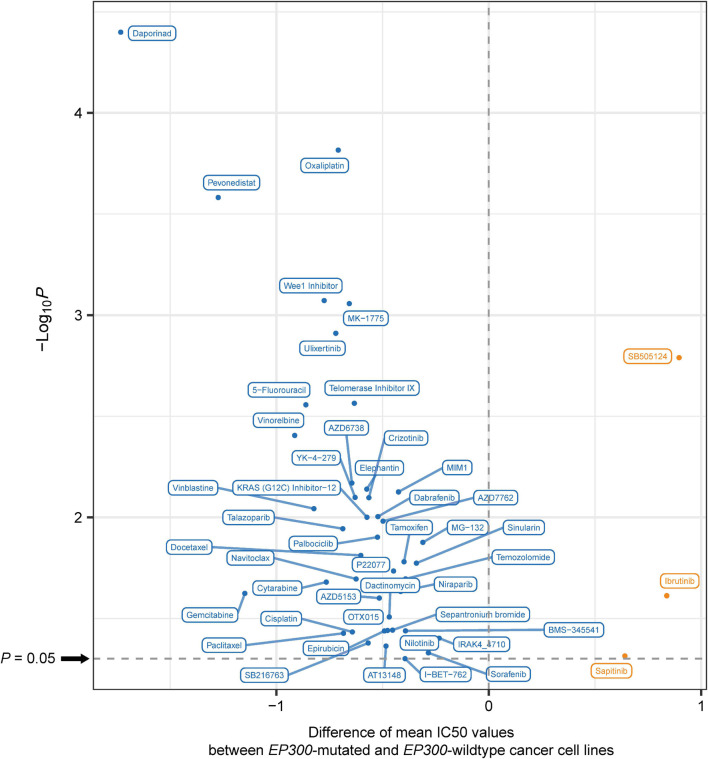
The compounds to which E1A binding protein p300 (*EP300*)-mutated and *EP300*-wild-type cancer cell lines have significantly different sensitivities. Blue indicates higher sensitivity (lower IC50 values) in *EP300*-mutated cell lines, and red indicates higher sensitivity (higher IC50 values) in *EP300*-wild-type cell lines (one-tailed Mann–Whitney *U* test, *p* < 0.05).

We analyzed the association between *EP300* mutations and drug sensitivity (IC50 values) of cancer cell lines to 192 antitumor compounds from the GDSC project.^7^ Interestingly, *EP300*-mutated cell lines had significantly lower IC50 values than *EP300*-wild-type cell lines for 44 compounds (one-tailed Mann–Whitney *U* test, *p* < 0.05) (Figure 5). It indicated that *EP300* mutations were associated with increased drug sensitivity of cancer cell lines to these compounds. These compounds mainly targeted pathways of Wnt, Receptor tyrosine kinase (RTK), phosphoinositide 3-kinase (PI3K)/mTOR, extracellular signal-regulated kinase (ERK)/MAPK, cell cycle, DNA replication, metabolism, genome integrity, apoptosis regulation, and chromatin. It is justified since our previous results have shown that most of these pathways associated with EP300. In contrast, for three compounds (sapitinib, ibrutinib, and SB505124), *EP300*-mutated cell lines had significantly higher IC50 values than those in *EP300*-wild-type cell lines. It indicated that *EP300* mutations were associated with reduced drug sensitivity of cancer cell lines to these compounds. Moreover, we analyzed the association between *EP300* mutations and drug sensitivity in individual types of cell lines. Likewise, we found that *EP300* mutations were associated with increased drug sensitivity of cancer cell lines to many compounds in individual cancer types (Supplementary Table 6). Overall, these results suggest that *EP300* mutations may enhance the sensitivity of cancers to many antitumor drugs.

## Discussion

**FIGURE 6:**
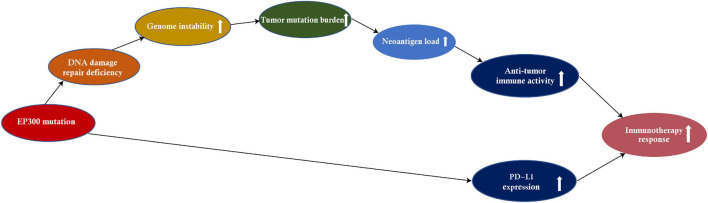
The mechanism by which the E1A binding protein p300 (*EP300*) mutation increases antitumor immunity and immunotherapeutic response in cancer.

This study investigated associations of *EP300* mutations with genome instability and tumor immunity by pan-cancer analysis of 11 cancer types. These cancer types included those most common cancers, such as lung, breast, colon, skin, and stomach cancers.^8^ We found that *EP300* mutations had significant associations with genome instability (e.g., increased TMB) and increased antitumor immunity in diverse cancers (Figure 6). Moreover, *EP300* mutations were associated with increased PD-L1 expression in diverse cancers. Because both high TMB and PD-L1 expression were associated with a more active response to ICIs (Ayers et al., 2017), *EP300*-mutated cancers would respond better to ICIs vs. *EP300*-wild-type cancers. This inference was evidenced in three melanoma cohorts receiving ICI therapy.

The underlying mechanism of *EP300* mutations in promoting genome instability could be that EP300 dysfunction caused by *EP300* mutations compromises its DDR function. Indeed, previous studies have shown that histone acetyltransferases play significant roles in DDR (Vidanes et al., 2005; van Attikum and Gasser, 2009; Cao et al., 2016). Our data also showed that *EP300* mutations were correlated with DDR deficiency, such as a high proportion of MSI cancers in the *EP300*-mutated subtype and co-mutation of *EP300* with DNA mismatch repair and DDR pathway genes. Furthermore, because of increased TMB to create more neoantigens due to DDR deficiency, *EP300*-mutated cancers displayed stronger antitumor immune activity.

We found that *EP300*-mutated cancers more highly expressed immune (such as cytokine–cytokine receptor interaction and Jak–STAT signaling), oncogenic (such as cell cycle), and DDR (such as p53 signaling) pathways (Figure 3). Again, elevated immune activity in *EP300*-mutated cancers suggests that this subtype could respond better to immunotherapy since the inflamed tumor immune microenvironment may promote the response to immunotherapy (Li and Wang, 2021). Elevated cell cycle activity in *EP300*-mutated cancers indicates that this subtype could have a favorable response to cell cycle inhibitors. In fact, by analyzing the association between *EP300* mutations and drug sensitivity of cancer cell lines, we found that *EP300*-mutated cancers were more responsive to several cell cycle inhibitors, including AZD7762, Wee1 inhibitor, RO-3306, palbociclib, BI-2536, MK-1775, dinaciclib, ribociclib, and MK-8776 (Figure 5).

## Conclusion

The *EP300* mutation correlates with heightened genome instability, antitumor immune activity, and immunotherapeutic response in cancer. Thus, the *EP300* mutation is a predictive biomarker for the response to immunotherapy, although more clinical data are needed to reinforce this reference.

## Data Availability Statement

The datasets presented in this study can be found in online repositories. The names of the repository/repositories and accession number(s) can be found in the article/Supplementary Material.

## Author Contributions

ZC contributed to software acquisition, validation, formal analysis, investigation, data curation, writing–review and editing, and funding acquisition. CC and LL contributed to software acquisition, validation, formal analysis, investigation, data curation, visualization, and writing–review and editing. TZ contributed to the formal analysis, resources, and investigation. XW contributed to the conceptualization, methodology, resources, investigation, writing–original draft, writing–review and editing, supervision, project administration, and funding acquisition. All authors contributed to the article and approved the submitted version.

## Conflict of Interest

The authors declare that the research was conducted in the absence of any commercial or financial relationships that could be construed as a potential conflict of interest.

## Publisher’s Note

All claims expressed in this article are solely those of the authors and do not necessarily represent those of their affiliated organizations, or those of the publisher, the editors and the reviewers. Any product that may be evaluated in this article, or claim that may be made by its manufacturer, is not guaranteed or endorsed by the publisher.

## Footnotes

1
https://cancergenome.nih.gov

2
https://portal.gdc.cancer.gov/

3
https://portals.broadinstitute.org/ccle/data

4
https://www.cancerrxgene.org

5
https://thebiogrid.org/

6
https://portals.broadinstitute.org/ccle

7
https://www.cancerrxgene.org

8
https://www.who.int/news-room/fact-sheets/detail/cancer
